# Supplementary material for: A fault-intrusion-tolerant system and deadline-aware algorithm for scheduling scientific workflow in the cloud
Source: PeerJ Comput Sci. 2021 Nov 2;7:e747. doi: 10.7717/peerj-cs.747 (PMC8576554; doi:10.7717/peerj-cs.747)
Supplement: Supplemental Information 6 [file peerj-cs-07-747-s006.zip › Myproject1.1/docs/index-files/index-18.html]

S-Index


JavaScript is disabled on your browser.


- Overview
- Package
- Class
- Use
- Tree
- Deprecated
- Index
- Help

- Prev Letter
- Next Letter

- Frames
- No Frames

- All Classes

A B C D E F G H I J L M N O P Q R S T U V W Z 


## S

sample() - Method in interface org.cloudbus.cloudsim.distributions.ContinuousDistribution
:   Sample the random number generator.

sample() - Method in class org.cloudbus.cloudsim.distributions.ExponentialDistr
:   Generate a new random number.

sample() - Method in class org.cloudbus.cloudsim.distributions.GammaDistr


sample() - Method in class org.cloudbus.cloudsim.distributions.LognormalDistr


sample() - Method in class org.cloudbus.cloudsim.distributions.LomaxDistribution


sample() - Method in class org.cloudbus.cloudsim.distributions.ParetoDistr


sample() - Method in class org.cloudbus.cloudsim.distributions.UniformDistr
:   Generate a new random number.

sample(Random, double, double) - Static method in class org.cloudbus.cloudsim.distributions.UniformDistr
:   Generates a new random number based on the number generator and values provided as
    parameters.

sample() - Method in class org.cloudbus.cloudsim.distributions.WeibullDistr


sample() - Method in class org.cloudbus.cloudsim.distributions.ZipfDistr
:   Generate a new random number.

SanStorage - Class in org.cloudbus.cloudsim
:   SANStorage represents a storage area network composed of a set of harddisks connected in a LAN.

SanStorage(double, double, double) - Constructor for class org.cloudbus.cloudsim.SanStorage
:   Creates a new SAN with a given capacity, latency, and bandwidth of the network connection.

SanStorage(String, double, double, double) - Constructor for class org.cloudbus.cloudsim.SanStorage
:   Creates a new SAN with a given capacity, latency, and bandwidth of the network connection.

saveAllocation() - Method in class org.cloudbus.cloudsim.power.PowerVmAllocationPolicyMigrationAbstract
:   Save allocation.

saveHistory(String) - Method in class org.cloudbus.cloudsim.UtilizationModelStochastic
:   Save history.

schedule(int, double, int, Object) - Method in class org.cloudbus.cloudsim.core.SimEntity
:   Send an event to another entity by id number, with data.

schedule(int, double, int) - Method in class org.cloudbus.cloudsim.core.SimEntity
:   Send an event to another entity by id number and with **no** data.

schedule(String, double, int, Object) - Method in class org.cloudbus.cloudsim.core.SimEntity
:   Send an event to another entity through a port with a given name, with data.

schedule(String, double, int) - Method in class org.cloudbus.cloudsim.core.SimEntity
:   Send an event to another entity through a port with a given name, with **no** data.

SCHEDULE\_NOW - Static variable in class org.cloudbus.cloudsim.core.CloudSimTags
:   Schedules an entity without any delay

scheduledBy() - Method in class org.cloudbus.cloudsim.core.SimEvent
:   Get the unique id number of the entity which scheduled this event.

scheduleFirst(int, double, int, Object) - Method in class org.cloudbus.cloudsim.core.SimEntity
:   Send a high priority event to another entity by id number, with data.

scheduleFirst(int, double, int) - Method in class org.cloudbus.cloudsim.core.SimEntity
:   Send a high priority event to another entity by id number and with **no** data.

scheduleFirst(String, double, int, Object) - Method in class org.cloudbus.cloudsim.core.SimEntity
:   Send a high priority event to another entity through a port with a given name, with data.

scheduleFirst(String, double, int) - Method in class org.cloudbus.cloudsim.core.SimEntity
:   Send a high priority event to another entity through a port with a given name, with **no**
    data.

scheduleFirstNow(int, int, Object) - Method in class org.cloudbus.cloudsim.core.SimEntity
:   Send a high priority event to another entity by id number, with data.

scheduleFirstNow(int, int) - Method in class org.cloudbus.cloudsim.core.SimEntity
:   Send a high priority event to another entity by id number and with **no** data.

scheduleFirstNow(String, int, Object) - Method in class org.cloudbus.cloudsim.core.SimEntity
:   Send a high priority event to another entity through a port with a given name, with data.

scheduleFirstNow(String, int) - Method in class org.cloudbus.cloudsim.core.SimEntity
:   Send a high priority event to another entity through a port with a given name, with **no**
    data.

scheduleNow(int, int, Object) - Method in class org.cloudbus.cloudsim.core.SimEntity
:   Send an event to another entity by id number, with data.

scheduleNow(int, int) - Method in class org.cloudbus.cloudsim.core.SimEntity
:   Send an event to another entity by id number and with **no** data.

scheduleNow(String, int, Object) - Method in class org.cloudbus.cloudsim.core.SimEntity
:   Send an event to another entity through a port with a given name, with data.

scheduleNow(String, int) - Method in class org.cloudbus.cloudsim.core.SimEntity
:   Send an event to another entity through a port with a given name, with **no** data.

SchedulerInterface - Interface in org.workflowsim.scheduler
:   The Scheduler interface

SCHEDULING\_INTERVAL - Static variable in class org.cloudbus.cloudsim.examples.power.Constants


seed - Static variable in class org.cloudbus.cloudsim.network.datacenter.NetworkConstants


select(int, Predicate) - Static method in class org.cloudbus.cloudsim.core.CloudSim
:   Selects an event matching a predicate.

selectEvent(Predicate) - Method in class org.cloudbus.cloudsim.core.SimEntity
:   Extract the first event matching a predicate waiting in the entity's deferred queue.

send(int, int, double, int, Object) - Static method in class org.cloudbus.cloudsim.core.CloudSim
:   Used to send an event from one entity to another.

send(int, double, int, Object) - Method in class org.cloudbus.cloudsim.core.SimEntity
:   Sends an event/message to another entity by delaying the simulation time from the
    current time, with a tag representing the event type.

send(int, double, int) - Method in class org.cloudbus.cloudsim.core.SimEntity
:   Sends an event/message to another entity by delaying the simulation time from the
    current time, with a tag representing the event type.

send(String, double, int, Object) - Method in class org.cloudbus.cloudsim.core.SimEntity
:   Sends an event/message to another entity by delaying the simulation time from the
    current time, with a tag representing the event type.

send(String, double, int) - Method in class org.cloudbus.cloudsim.core.SimEntity
:   Sends an event/message to another entity by delaying the simulation time from the
    current time, with a tag representing the event type.

SEND - Static variable in class org.cloudbus.cloudsim.core.SimEvent


sendFirst(int, int, double, int, Object) - Static method in class org.cloudbus.cloudsim.core.CloudSim
:   Used to send an event from one entity to another, with priority in the queue.

sendNow(int, int, Object) - Method in class org.cloudbus.cloudsim.core.SimEntity
:   Sends an event/message to another entity by delaying the simulation time from the
    current time, with a tag representing the event type.

sendNow(int, int) - Method in class org.cloudbus.cloudsim.core.SimEntity
:   Sends an event/message to another entity by delaying the simulation time from the
    current time, with a tag representing the event type.

sendNow(String, int, Object) - Method in class org.cloudbus.cloudsim.core.SimEntity
:   Sends an event/message to another entity by delaying the simulation time from the
    current time, with a tag representing the event type.

sendNow(String, int) - Method in class org.cloudbus.cloudsim.core.SimEntity
:   Sends an event/message to another entity by delaying the simulation time from the
    current time, with a tag representing the event type.

setActive(boolean) - Method in class org.cloudbus.cloudsim.HostStateHistoryEntry
:   Sets the active.

setAllocatedMips(double) - Method in class org.cloudbus.cloudsim.HostStateHistoryEntry
:   Sets the allocated mips.

setAllocatedMips(double) - Method in class org.cloudbus.cloudsim.VmStateHistoryEntry
:   Sets the allocated mips.

setAllocationPolicy(int) - Method in class org.cloudbus.cloudsim.DatacenterCharacteristics
:   Sets the allocation policy.

setAppCloudletList(List<T>) - Method in class org.cloudbus.cloudsim.network.datacenter.NetDatacenterBroker


setArchitecture(String) - Method in class org.cloudbus.cloudsim.DatacenterCharacteristics
:   Sets the architecture.

setAvailableBw(long) - Method in class org.cloudbus.cloudsim.provisioners.BwProvisioner
:   Sets the available bw.

setAvailableMips(double) - Method in class org.cloudbus.cloudsim.provisioners.PeProvisioner
:   Sets the available MIPS.

setAvailableMips(double) - Method in class org.cloudbus.cloudsim.VmScheduler
:   Sets the free mips.

setAvailableRam(int) - Method in class org.cloudbus.cloudsim.provisioners.RamProvisioner
:   Sets the available ram.

setAvgSeekTime(double) - Method in class org.cloudbus.cloudsim.HarddriveStorage
:   Sets the average seek time of the storage in seconds.

setAvgSeekTime(double, ContinuousDistribution) - Method in class org.cloudbus.cloudsim.HarddriveStorage
:   Sets the average seek time and a new generator of seek times in seconds.

setBandwidth(String, double) - Method in class org.workflowsim.ClusterStorage
:   Sets the bandwidth between this storage to the destination storage

setBeingInstantiated(boolean) - Method in class org.cloudbus.cloudsim.Vm
:   Sets the being instantiated.

setBroker(DatacenterBroker) - Method in class org.cloudbus.cloudsim.examples.CloudSimExample8.GlobalBroker


setBw(long) - Method in class org.cloudbus.cloudsim.provisioners.BwProvisioner
:   Sets the bw.

setBw(long) - Method in class org.cloudbus.cloudsim.Vm
:   Sets the amount of bandwidth.

setBwProvisioner(BwProvisioner) - Method in class org.cloudbus.cloudsim.Host
:   Sets the bw provisioner.

setBwTable(Map<String, Long>) - Method in class org.cloudbus.cloudsim.provisioners.BwProvisionerSimple
:   Sets the bw table.

setCacheCurrentRequestedMips(List<Double>) - Method in class org.cloudbus.cloudsim.CloudletSchedulerDynamicWorkload
:   Sets the cache current requested mips.

setCachePreviousTime(double) - Method in class org.cloudbus.cloudsim.CloudletSchedulerDynamicWorkload
:   Sets the cache previous time.

setCharacteristics(DatacenterCharacteristics) - Method in class org.cloudbus.cloudsim.Datacenter
:   Sets the characteristics.

setChecksum(int) - Method in class org.cloudbus.cloudsim.File
:   Sets the checksum of this file

setChecksum(int) - Method in class org.cloudbus.cloudsim.FileAttribute
:   Sets the checksum of this file

setChildList(List) - Method in class org.workflowsim.Task
:   Sets the child list of the task

setClassType(int) - Method in class org.cloudbus.cloudsim.Cloudlet
:   Sets the classType or priority of this Cloudlet for scheduling on a resource.

setCloudletExecList(List<T>) - Method in class org.cloudbus.cloudsim.CloudletSchedulerSpaceShared
:   Sets the cloudlet exec list.

setCloudletExecList(List<T>) - Method in class org.cloudbus.cloudsim.CloudletSchedulerTimeShared
:   Sets the cloudlet exec list.

setCloudletExecList(List<T>) - Method in class org.cloudbus.cloudsim.network.datacenter.NetworkCloudletSpaceSharedScheduler
:   Sets the cloudlet exec list.

setCloudletFinishedList(List<T>) - Method in class org.cloudbus.cloudsim.CloudletSchedulerSpaceShared
:   Sets the cloudlet finished list.

setCloudletFinishedList(List<T>) - Method in class org.cloudbus.cloudsim.CloudletSchedulerTimeShared
:   Sets the cloudlet finished list.

setCloudletFinishedList(List<T>) - Method in class org.cloudbus.cloudsim.network.datacenter.NetworkCloudletSpaceSharedScheduler
:   Sets the cloudlet finished list.

setCloudletFinishedSoFar(long) - Method in class org.cloudbus.cloudsim.Cloudlet
:   Sets the length of this Cloudlet that has been executed so far.

setCloudletLength(long) - Method in class org.cloudbus.cloudsim.Cloudlet
:   Sets the length or size (in MI) of this Cloudlet to be executed in a CloudResource.

setCloudletList(List<T>) - Method in class org.cloudbus.cloudsim.DatacenterBroker
:   Sets the cloudlet list.

setCloudletList(List<Cloudlet>) - Method in class org.cloudbus.cloudsim.examples.CloudSimExample8.GlobalBroker


setCloudletList(List<T>) - Method in class org.cloudbus.cloudsim.network.datacenter.NetDatacenterBroker
:   Sets the cloudlet list.

setCloudletList(List) - Method in class org.workflowsim.scheduler.BaseScheduler
:   Sets the job list.

setCloudletList(List) - Method in interface org.workflowsim.scheduler.SchedulerInterface
:   Sets the job list.

setCloudletPausedList(List<T>) - Method in class org.cloudbus.cloudsim.CloudletSchedulerSpaceShared
:   Sets the cloudlet paused list.

setCloudletPausedList(List<T>) - Method in class org.cloudbus.cloudsim.CloudletSchedulerTimeShared
:   Sets the cloudlet paused list.

setCloudletPausedList(List<T>) - Method in class org.cloudbus.cloudsim.network.datacenter.NetworkCloudletSpaceSharedScheduler
:   Sets the cloudlet paused list.

setCloudletReceivedList(List<T>) - Method in class org.cloudbus.cloudsim.DatacenterBroker
:   Sets the cloudlet received list.

setCloudletReceivedList(List<T>) - Method in class org.cloudbus.cloudsim.network.datacenter.NetDatacenterBroker
:   Sets the cloudlet received list.

setCloudletScheduler(CloudletScheduler) - Method in class org.cloudbus.cloudsim.Vm
:   Sets the vm scheduler.

setCloudletStatus(int) - Method in class org.cloudbus.cloudsim.Cloudlet
:   Sets the status code of this Cloudlet.

setCloudletStatus(int) - Method in class org.cloudbus.cloudsim.ResCloudlet
:   Sets the Cloudlet status.

setCloudletSubmitted(double) - Method in class org.cloudbus.cloudsim.power.PowerDatacenter
:   Sets the cloudlet submited.

setCloudletSubmittedList(List<T>) - Method in class org.cloudbus.cloudsim.DatacenterBroker
:   Sets the cloudlet submitted list.

setCloudletSubmittedList(List<T>) - Method in class org.cloudbus.cloudsim.network.datacenter.NetDatacenterBroker
:   Sets the cloudlet submitted list.

setComment(String) - Method in class org.cloudbus.cloudsim.util.WorkloadFileReader
:   Identifies the start of a comment line.

setConstant(double) - Method in class org.cloudbus.cloudsim.power.models.PowerModelCubic
:   Sets the constant.

setConstant(double) - Method in class org.cloudbus.cloudsim.power.models.PowerModelLinear
:   Sets the constant.

setConstant(double) - Method in class org.cloudbus.cloudsim.power.models.PowerModelSqrt
:   Sets the constant.

setConstant(double) - Method in class org.cloudbus.cloudsim.power.models.PowerModelSquare
:   Sets the constant.

setCost(double) - Method in class org.cloudbus.cloudsim.File
:   Sets the cost associated with this file

setCost(double) - Method in class org.cloudbus.cloudsim.FileAttribute
:   Sets the cost associated with this file

setCostPerBw(double) - Method in class org.cloudbus.cloudsim.DatacenterCharacteristics
:   Sets cost to use bw cost to use bw.

setCostPerMem(double) - Method in class org.cloudbus.cloudsim.DatacenterCharacteristics
:   Sets cost to use memory.

setCostPerSecond(double) - Method in class org.cloudbus.cloudsim.DatacenterCharacteristics
:   Sets the cost per second.

setCostPerStorage(double) - Method in class org.cloudbus.cloudsim.DatacenterCharacteristics
:   Sets cost to use storage.

setCreationTime(long) - Method in class org.cloudbus.cloudsim.FileAttribute
:   Sets the file creation time (in millisecond)

setCurrentAllocatedBw(long) - Method in class org.cloudbus.cloudsim.Vm
:   Sets the current allocated bw.

setCurrentAllocatedMips(List<Double>) - Method in class org.cloudbus.cloudsim.Vm
:   Sets the current allocated mips.

setCurrentAllocatedRam(int) - Method in class org.cloudbus.cloudsim.Vm
:   Sets the current allocated ram.

setCurrentAllocatedSize(long) - Method in class org.cloudbus.cloudsim.Vm
:   Sets the current allocated size.

setCurrentMipsShare(List<Double>) - Method in class org.cloudbus.cloudsim.CloudletScheduler
:   Sets the current mips share.

setDatacenter(Datacenter) - Method in class org.cloudbus.cloudsim.Host
:   Sets the data center.

setDatacenterCharacteristicsList(Map<Integer, DatacenterCharacteristics>) - Method in class org.cloudbus.cloudsim.DatacenterBroker
:   Sets the datacenter characteristics list.

setDatacenterCharacteristicsList(Map<Integer, DatacenterCharacteristics>) - Method in class org.cloudbus.cloudsim.network.datacenter.NetDatacenterBroker
:   Sets the datacenter characteristics list.

setDatacenterIdsList(List<Integer>) - Method in class org.cloudbus.cloudsim.DatacenterBroker
:   Sets the datacenter ids list.

setDatacenterIdsList(List<Integer>) - Method in class org.cloudbus.cloudsim.network.datacenter.NetDatacenterBroker
:   Sets the datacenter ids list.

setDatacenterRequestedIdsList(List<Integer>) - Method in class org.cloudbus.cloudsim.DatacenterBroker
:   Sets the datacenter requested ids list.

setDatacenterRequestedIdsList(List<Integer>) - Method in class org.cloudbus.cloudsim.network.datacenter.NetDatacenterBroker
:   Sets the datacenter requested ids list.

setDepth(int) - Method in class org.workflowsim.Task
:   Sets the depth of the task

setDestId(int) - Method in class org.cloudbus.cloudsim.InfoPacket
:   Sets the destination ID for this packet.

setDestination(int) - Method in class org.cloudbus.cloudsim.core.SimEvent
:   Set the destination entity of this event.

setDisabled(boolean) - Static method in class org.cloudbus.cloudsim.Log
:   Sets the disable output flag.

setDisableMigrations(boolean) - Method in class org.cloudbus.cloudsim.power.PowerDatacenter
:   Sets the disable migrations.

setEnableOutput(boolean) - Method in class org.cloudbus.cloudsim.examples.power.RunnerAbstract
:   Sets the enable output.

setEndWaitingTime(double) - Method in class org.cloudbus.cloudsim.core.SimEvent
:   Used to set the time at which this event finished waiting in the event

setEventBuffer(SimEvent) - Method in class org.cloudbus.cloudsim.core.SimEntity
:   Sets the event buffer.

setExecParam(double, double) - Method in class org.cloudbus.cloudsim.Cloudlet
:   Sets this Cloudlet's execution parameters.

setExecParam(double, double) - Method in class org.cloudbus.cloudsim.ResCloudlet
:   Sets this Cloudlet's execution parameters.

setExecStartTime(double) - Method in class org.cloudbus.cloudsim.Cloudlet
:   Sets the execution start time of this Cloudlet inside a CloudResource.

setFailed(String, boolean) - Method in class org.cloudbus.cloudsim.Host
:   Sets the PEs of this machine to a FAILED status.

setFailed(boolean) - Method in class org.cloudbus.cloudsim.Host
:   Sets the PEs of this machine to a FAILED status.

setFallbackPolicy(PowerVmSelectionPolicy) - Method in class org.cloudbus.cloudsim.power.PowerVmSelectionPolicyMaximumCorrelation
:   Sets the fallback policy.

setFallbackVmAllocationPolicy(PowerVmAllocationPolicyMigrationAbstract) - Method in class org.cloudbus.cloudsim.power.PowerVmAllocationPolicyMigrationInterQuartileRange
:   Sets the fallback vm allocation policy.

setFallbackVmAllocationPolicy(PowerVmAllocationPolicyMigrationAbstract) - Method in class org.cloudbus.cloudsim.power.PowerVmAllocationPolicyMigrationLocalRegression
:   Sets the fallback vm allocation policy.

setFallbackVmAllocationPolicy(PowerVmAllocationPolicyMigrationAbstract) - Method in class org.cloudbus.cloudsim.power.PowerVmAllocationPolicyMigrationMedianAbsoluteDeviation
:   Sets the fallback vm allocation policy.

setField(int, int, int, int, int) - Method in class org.cloudbus.cloudsim.util.WorkloadFileReader
:   Tells this class what to look in the trace file.

setFile(String, File) - Static method in class org.workflowsim.utils.ReplicaCatalog
:   Adds a file name and the associated file object

setFileList(List<File>) - Method in class org.workflowsim.Task
:   Sets a file list

setFileSize(int) - Method in class org.cloudbus.cloudsim.File
:   Sets the file size (in MBytes)

setFileSize(int) - Method in class org.cloudbus.cloudsim.FileAttribute
:   Sets the file size (in MBytes)

setFinishTime(double) - Method in class org.cloudbus.cloudsim.ResCloudlet
:   Sets the finish time for this Cloudlet.

setFreePes(List<Integer>) - Method in class org.cloudbus.cloudsim.network.datacenter.NetworkVmAllocationPolicy
:   Sets the free pes.

setFreePes(List<Integer>) - Method in class org.cloudbus.cloudsim.VmAllocationPolicySimple
:   Sets the free pes.

setFreePes(List<Pe>) - Method in class org.cloudbus.cloudsim.VmSchedulerSpaceShared
:   Sets the free pes vector.

setHistory(Map<Double, Double>) - Method in class org.cloudbus.cloudsim.UtilizationModelStochastic
:   Sets the history.

setHost(Host) - Method in class org.cloudbus.cloudsim.Vm
:   Sets the host that runs this VM.

setHostList(List<T>) - Method in class org.cloudbus.cloudsim.DatacenterCharacteristics
:   Sets the host list.

setHostList(List<? extends Host>) - Method in class org.cloudbus.cloudsim.VmAllocationPolicy
:   Sets the host list.

setId(int) - Method in class org.cloudbus.cloudsim.core.SimEntity
:   Sets the id.

setId(int) - Method in class org.cloudbus.cloudsim.DatacenterCharacteristics
:   Sets the id.

setId(int) - Method in class org.cloudbus.cloudsim.Host
:   Sets the id.

setId(int) - Method in class org.cloudbus.cloudsim.Pe
:   Sets the id.

setId(int) - Method in class org.cloudbus.cloudsim.Vm
:   Sets the id.

setImpact(double) - Method in class org.workflowsim.Task
:   Sets the impact factor

setImpactFafctor(double) - Method in class org.workflowsim.clustering.TaskSet
:   Sets the impact factor

setInMigration(boolean) - Method in class org.cloudbus.cloudsim.Vm
:   Sets the in migration.

setInMigration(boolean) - Method in class org.cloudbus.cloudsim.VmStateHistoryEntry
:   Sets the in migration.

setJobList(List<Job>) - Method in class org.workflowsim.ClusteringEngine
:   Sets the job list.

setLast(int) - Method in class org.cloudbus.cloudsim.InfoPacket
:   Sets an entity ID from the last hop that this packet has traversed.

setLast(int) - Method in interface org.cloudbus.cloudsim.Packet
:   Sets an entity ID from the last hop that this packet has traversed.

setLastProcessTime(double) - Method in class org.cloudbus.cloudsim.Datacenter
:   Sets the last process time.

setLatency(double) - Method in class org.cloudbus.cloudsim.HarddriveStorage
:   Sets the latency of this harddrive in seconds.

setLinkDC(NetworkDatacenter) - Method in class org.cloudbus.cloudsim.network.datacenter.NetDatacenterBroker


setMachineAndPeId(int, int) - Method in class org.cloudbus.cloudsim.ResCloudlet
:   Sets the machine and Pe (Processing Element) ID.

setMasterCopy(boolean) - Method in class org.cloudbus.cloudsim.File
:   Marks this file as a master copy or replica

setMasterCopy(boolean) - Method in class org.cloudbus.cloudsim.FileAttribute
:   Marks this file as a master copy or replica

setMaxPower(double) - Method in class org.cloudbus.cloudsim.power.models.PowerModelCubic
:   Sets the max power.

setMaxPower(double) - Method in class org.cloudbus.cloudsim.power.models.PowerModelLinear
:   Sets the max power.

setMaxPower(double) - Method in class org.cloudbus.cloudsim.power.models.PowerModelSqrt
:   Sets the max power.

setMaxPower(double) - Method in class org.cloudbus.cloudsim.power.models.PowerModelSquare
:   Sets the max power.

setMaxTransferRate(int) - Method in class org.cloudbus.cloudsim.HarddriveStorage
:   Sets the maximum transfer rate of this storage system in MB/sec.

setMaxTransferRate(int) - Method in interface org.cloudbus.cloudsim.Storage
:   Sets the maximum transfer rate of this storage system in MByte/sec.

setMigrationCount(int) - Method in class org.cloudbus.cloudsim.power.PowerDatacenter
:   Sets the migration count.

setMips(double) - Method in class org.cloudbus.cloudsim.CloudletSchedulerDynamicWorkload
:   Sets the mips.

setMips(double) - Method in class org.cloudbus.cloudsim.Pe
:   Sets the MIPS Rating of this Pe.

setMips(double) - Method in class org.cloudbus.cloudsim.provisioners.PeProvisioner
:   Sets the MIPS.

setMips(double) - Method in class org.cloudbus.cloudsim.Vm
:   Sets the mips.

setMipsMap(Map<String, List<Double>>) - Method in class org.cloudbus.cloudsim.VmScheduler
:   Sets the mips map.

setMipsMapRequested(Map<String, List<Double>>) - Method in class org.cloudbus.cloudsim.VmSchedulerTimeShared
:   Sets the mips map requested.

setName(String) - Method in class org.cloudbus.cloudsim.File
:   Sets the file name

setName(String) - Method in class org.cloudbus.cloudsim.FileAttribute
:   Sets the file name

setNetServiceLevel(int) - Method in class org.cloudbus.cloudsim.Cloudlet
:   Sets the network service level for sending this cloudlet over a network.

setNetServiceType(int) - Method in class org.cloudbus.cloudsim.InfoPacket
:   Sets the network service type of this packet.

setNetServiceType(int) - Method in interface org.cloudbus.cloudsim.Packet
:   Sets the network service type of this packet.

setNumberOfPes(int) - Method in class org.cloudbus.cloudsim.Cloudlet
:   Sets the number of PEs required to run this Cloudlet.

setNumberOfPes(int) - Method in class org.cloudbus.cloudsim.CloudletSchedulerDynamicWorkload
:   Sets the pes number.

setNumberOfPes(int) - Method in class org.cloudbus.cloudsim.Vm
:   Sets the number of pes.

setOriginalPingSize(long) - Method in class org.cloudbus.cloudsim.InfoPacket
:   Sets original size of ping request.

setOs(String) - Method in class org.cloudbus.cloudsim.DatacenterCharacteristics
:   Sets the os.

setOutput(OutputStream) - Static method in class org.cloudbus.cloudsim.Log
:   Sets the output.

setOwnerName(String) - Method in class org.cloudbus.cloudsim.File
:   Sets the owner name of this file

setOwnerName(String) - Method in class org.cloudbus.cloudsim.FileAttribute
:   Sets the owner name of this file

setParentList(List) - Method in class org.workflowsim.Task
:   Sets the parent list of the task

setPeAllocationMap(Map<String, List<Pe>>) - Method in class org.cloudbus.cloudsim.VmSchedulerSpaceShared
:   Sets the pe allocation map.

setPeList(List<T>) - Method in class org.cloudbus.cloudsim.Host
:   Sets the pe list.

setPeList(List<T>) - Method in class org.cloudbus.cloudsim.VmScheduler
:   Sets the vm list.

setPeMap(Map<String, List<Pe>>) - Method in class org.cloudbus.cloudsim.VmScheduler
:   Sets the pe map.

setPeProvisioner(PeProvisioner) - Method in class org.cloudbus.cloudsim.Pe
:   Sets the pe provisioner.

setPesInUse(int) - Method in class org.cloudbus.cloudsim.VmSchedulerTimeShared
:   Sets the pes in use.

setPeStatus(int, int, int) - Method in class org.cloudbus.cloudsim.DatacenterCharacteristics
:   Sets the particular Pe status on a Machine.

setPeStatus(int, int) - Method in class org.cloudbus.cloudsim.Host
:   Sets the particular Pe status on this Machine.

setPeStatus(List<T>, int, int, int) - Static method in class org.cloudbus.cloudsim.lists.HostList
:   Sets the particular Pe status on a Machine.

setPeStatus(List<T>, int, int) - Static method in class org.cloudbus.cloudsim.lists.PeList
:   Sets the Pe status.

setPeTable(Map<String, ? extends List<Double>>) - Method in class org.cloudbus.cloudsim.provisioners.PeProvisionerSimple
:   Sets the pe table.

setPower(double) - Method in class org.cloudbus.cloudsim.power.PowerDatacenter
:   Sets the power.

setPowerModel(PowerModel) - Method in class org.cloudbus.cloudsim.power.PowerHost
:   Sets the power model.

setPreviousTime(double) - Method in class org.cloudbus.cloudsim.CloudletScheduler
:   Sets the previous time.

setPreviousTime(double) - Method in class org.cloudbus.cloudsim.power.PowerVm
:   Sets the previous time.

setPreviousUtilizationMips(double) - Method in class org.cloudbus.cloudsim.HostDynamicWorkload
:   Sets the previous utilization mips.

setPriority(int) - Method in class org.workflowsim.Task
:   Sets the priority of the task

setRam(int) - Method in class org.cloudbus.cloudsim.provisioners.RamProvisioner
:   Sets the ram.

setRam(int) - Method in class org.cloudbus.cloudsim.Vm
:   Sets the amount of ram.

setRamProvisioner(RamProvisioner) - Method in class org.cloudbus.cloudsim.Host
:   Sets the ram provisioner.

setRamTable(Map<String, Integer>) - Method in class org.cloudbus.cloudsim.provisioners.RamProvisionerSimple
:   Sets the ram table.

setRandomGenerator(Random) - Method in class org.cloudbus.cloudsim.UtilizationModelStochastic
:   Sets the random generator.

setReadOnly(boolean) - Method in class org.cloudbus.cloudsim.File
:   Marks this file as a read only or not

setReadOnly(boolean) - Method in class org.cloudbus.cloudsim.FileAttribute
:   Marks this file as a read only or not

setRegionalCisName(String) - Method in class org.cloudbus.cloudsim.Datacenter
:   Sets the regional cis name.

setRegistrationID(int) - Method in class org.cloudbus.cloudsim.File
:   Sets the file registration ID (published by a Replica Catalogue entity)

setRegistrationId(int) - Method in class org.cloudbus.cloudsim.FileAttribute
:   Sets the file registration ID (published by a Replica Catalogue entity)

setRequestedMips(double) - Method in class org.cloudbus.cloudsim.HostStateHistoryEntry
:   Sets the requested mips.

setRequestedMips(double) - Method in class org.cloudbus.cloudsim.VmStateHistoryEntry
:   Sets the requested mips.

setRequiredFiles(List<String>) - Method in class org.cloudbus.cloudsim.Cloudlet
:   Sets the required files.

setReservationId(int) - Method in class org.cloudbus.cloudsim.Cloudlet
:   Sets the id of the reservation made for this cloudlet.

setResourceID(int) - Method in class org.cloudbus.cloudsim.File
:   Sets the resource ID that stores this file

setResourceID(int) - Method in class org.cloudbus.cloudsim.FileAttribute
:   Sets the resource ID that stores this file

setResourceParameter(int, double) - Method in class org.cloudbus.cloudsim.Cloudlet
:   Sets the resource parameters for which this Cloudlet is going to be executed.

setResourceParameter(int, double, double) - Method in class org.cloudbus.cloudsim.Cloudlet
:   Sets the resource parameters for which this Cloudlet is going to be executed.

setSafetyParameter(double) - Method in class org.cloudbus.cloudsim.power.PowerVmAllocationPolicyMigrationInterQuartileRange
:   Sets the safety parameter.

setSafetyParameter(double) - Method in class org.cloudbus.cloudsim.power.PowerVmAllocationPolicyMigrationLocalRegression


setSafetyParameter(double) - Method in class org.cloudbus.cloudsim.power.PowerVmAllocationPolicyMigrationMedianAbsoluteDeviation
:   Sets the safety parameter.

setSchedulingInterval(double) - Method in class org.cloudbus.cloudsim.Datacenter
:   Sets the scheduling interval.

setSchedulingInterval(double) - Method in class org.cloudbus.cloudsim.power.PowerVm
:   Sets the scheduling interval.

setSchedulingInterval(double) - Method in class org.cloudbus.cloudsim.power.PowerVmAllocationPolicyMigrationLocalRegression
:   Sets the scheduling interval.

setSchedulingInterval(double) - Method in class org.cloudbus.cloudsim.UtilizationModelPlanetLabInMemory
:   Sets the scheduling interval.

setSeed(long) - Method in class org.cloudbus.cloudsim.distributions.UniformDistr
:   Set the random number generator's seed.

setSerial(long) - Method in class org.cloudbus.cloudsim.core.SimEvent


setSize(long) - Method in class org.cloudbus.cloudsim.InfoPacket
:   Sets the size of this packet.

setSize(long) - Method in interface org.cloudbus.cloudsim.Packet
:   Sets the size of this packet

setSize(long) - Method in class org.cloudbus.cloudsim.Vm
:   Sets the amount of storage.

setSource(int) - Method in class org.cloudbus.cloudsim.core.SimEvent
:   Set the source entity of this event.

setState(int) - Method in class org.cloudbus.cloudsim.core.SimEntity
:   Sets the state.

setState(int) - Method in class org.workflowsim.CondorVM
:   Sets the state of the task

setStaticPower(double) - Method in class org.cloudbus.cloudsim.power.models.PowerModelCubic
:   Sets the static power.

setStaticPower(double) - Method in class org.cloudbus.cloudsim.power.models.PowerModelLinear
:   Sets the static power.

setStaticPower(double) - Method in class org.cloudbus.cloudsim.power.models.PowerModelSqrt
:   Sets the static power.

setStaticPower(double) - Method in class org.cloudbus.cloudsim.power.models.PowerModelSquare
:   Sets the static power.

setStatus(int) - Method in class org.cloudbus.cloudsim.Pe
:   Sets Pe status to either Pe.FREE or Pe.BUSY

setStatusBusy() - Method in class org.cloudbus.cloudsim.Pe
:   Sets Pe status to busy, meaning it is already executing Cloudlets.

setStatusFailed(List<T>, String, int, boolean) - Static method in class org.cloudbus.cloudsim.lists.PeList
:   Sets the status of PEs of this machine to FAILED.

setStatusFailed(List<T>, boolean) - Static method in class org.cloudbus.cloudsim.lists.PeList
:   Sets the status of PEs of this machine to FAILED.

setStatusFailed() - Method in class org.cloudbus.cloudsim.Pe
:   Sets this Pe to FAILED.

setStatusFree() - Method in class org.cloudbus.cloudsim.Pe
:   Sets Pe status to free, meaning it is available for processing.

setStorage(long) - Method in class org.cloudbus.cloudsim.Host
:   Sets the storage.

setStorageList(List<Storage>) - Method in class org.cloudbus.cloudsim.Datacenter
:   Sets the storage list.

setSubmissionTime(double) - Method in class org.cloudbus.cloudsim.Cloudlet
:   Sets the submission or arrival time of this Cloudlet into a CloudResource.

setTag(int) - Method in class org.cloudbus.cloudsim.InfoPacket
:   Sets the tag of this packet.

setTaskList(List<Task>) - Method in class org.workflowsim.clustering.BasicClustering
:   Sets the task list

setTaskList(List<Task>) - Method in interface org.workflowsim.clustering.ClusteringInterface
:   set the task list.

setTaskList(List<Task>) - Method in class org.workflowsim.ClusteringEngine
:   Sets the task list.

setTaskList(List) - Method in class org.workflowsim.Job
:   Sets the list of the tasks

setTaskList(List) - Method in class org.workflowsim.planning.BasePlanner
:   Sets the job list.

setTaskList(List) - Method in interface org.workflowsim.planning.PlannerInterface
:   Sets the task list.

setTaskList(List<Task>) - Method in class org.workflowsim.WorkflowParser
:   Sets the task list

setTaskList(List<Task>) - Method in class org.workflowsim.WorkflowPlanner
:   Sets the task list.

setTaskReceivedList(List<Task>) - Method in class org.workflowsim.ClusteringEngine
:   Sets the task received list.

setTaskSubmittedList(List<Task>) - Method in class org.workflowsim.ClusteringEngine
:   Sets the tasks submitted list.

setTime(double) - Method in class org.cloudbus.cloudsim.HostStateHistoryEntry
:   Sets the time.

setTime(double) - Method in class org.cloudbus.cloudsim.VmStateHistoryEntry
:   Sets the time.

setTimeZone(double) - Method in class org.cloudbus.cloudsim.DatacenterCharacteristics
:   Sets the time zone.

setTotalMips(double) - Method in class org.cloudbus.cloudsim.CloudletSchedulerDynamicWorkload
:   Sets the total mips.

setTransactionTime(double) - Method in class org.cloudbus.cloudsim.File
:   Sets the current transaction time (in second) of this file.

setType(int) - Method in class org.cloudbus.cloudsim.File
:   Sets the file type (e.g.

setType(int) - Method in class org.cloudbus.cloudsim.FileAttribute
:   Sets the file type (e.g.

setType(String) - Method in class org.workflowsim.Task
:   Sets the type of the task

setUid(String) - Method in class org.cloudbus.cloudsim.Vm
:   Sets the uid.

setUnderAllocatedMips(Map<String, Double>) - Method in class org.cloudbus.cloudsim.CloudletSchedulerDynamicWorkload
:   Sets the under allocated mips.

setUpdateTime(double) - Method in class org.cloudbus.cloudsim.File
:   Sets the last update time of this file (in seconds)  
    NOTE: This time is relative to the start time.

setUpdateTime(double) - Method in class org.cloudbus.cloudsim.FileAttribute
:   Sets the last update time of this file (in seconds)  
    NOTE: This time is relative to the start time.

setUsedPes(Map<String, Integer>) - Method in class org.cloudbus.cloudsim.network.datacenter.NetworkVmAllocationPolicy
:   Sets the used pes.

setUsedPes(Map<String, Integer>) - Method in class org.cloudbus.cloudsim.VmAllocationPolicySimple
:   Sets the used pes.

setUserId(int) - Method in class org.cloudbus.cloudsim.Cloudlet
:   Sets the user or owner ID of this Cloudlet.

setUserId(int) - Method in class org.cloudbus.cloudsim.Vm
:   Sets the user id.

setUtilizationMips(double) - Method in class org.cloudbus.cloudsim.HostDynamicWorkload
:   Sets the utilization mips.

setUtilizationModelBw(UtilizationModel) - Method in class org.cloudbus.cloudsim.Cloudlet
:   Sets the utilization model bw.

setUtilizationModelCpu(UtilizationModel) - Method in class org.cloudbus.cloudsim.Cloudlet
:   Sets the utilization model cpu.

setUtilizationModelRam(UtilizationModel) - Method in class org.cloudbus.cloudsim.Cloudlet
:   Sets the utilization model ram.

setUtilizationThreshold(double) - Method in class org.cloudbus.cloudsim.power.PowerVmAllocationPolicyMigrationStaticThreshold
:   Sets the utilization threshold.

setVmAllocationPolicy(VmAllocationPolicy) - Method in class org.cloudbus.cloudsim.Datacenter
:   Sets the vm allocation policy.

setVmId(int) - Method in class org.cloudbus.cloudsim.Cloudlet
:   Sets the ID of the VM that will run this Cloudlet.

setVmList(List<T>) - Method in class org.cloudbus.cloudsim.Datacenter
:   Sets the vm list.

setVmList(List<T>) - Method in class org.cloudbus.cloudsim.DatacenterBroker
:   Sets the vm list.

setVmList(List<Vm>) - Method in class org.cloudbus.cloudsim.examples.CloudSimExample8.GlobalBroker


setVmList(List<T>) - Method in class org.cloudbus.cloudsim.network.datacenter.NetDatacenterBroker
:   Sets the vm list.

setVmList(List) - Method in class org.workflowsim.planning.BasePlanner
:   Sets the vm list

setVmList(List) - Method in interface org.workflowsim.planning.PlannerInterface
:   Sets the vm list.

setVmList(List) - Method in class org.workflowsim.scheduler.BaseScheduler
:   Sets the vm list

setVmList(List) - Method in interface org.workflowsim.scheduler.SchedulerInterface
:   Sets the vm list.

setVmm(String) - Method in class org.cloudbus.cloudsim.DatacenterCharacteristics
:   Sets the vmm.

setVmm(String) - Method in class org.cloudbus.cloudsim.Vm
:   Sets the VMM.

setVmNum(int) - Static method in class org.workflowsim.utils.Parameters
:   Sets the vm number

setVmsAcks(int) - Method in class org.cloudbus.cloudsim.DatacenterBroker
:   Sets the vms acks.

setVmsAcks(int) - Method in class org.cloudbus.cloudsim.network.datacenter.NetDatacenterBroker
:   Sets the vms acks.

setVmScheduler(VmScheduler) - Method in class org.cloudbus.cloudsim.Host
:   Sets the VM scheduler.

setVmsCreatedList(List<T>) - Method in class org.cloudbus.cloudsim.DatacenterBroker
:   Sets the vm list.

setVmsCreatedList(List<T>) - Method in class org.cloudbus.cloudsim.network.datacenter.NetDatacenterBroker
:   Sets the vm list.

setVmsDestroyed(int) - Method in class org.cloudbus.cloudsim.DatacenterBroker
:   Sets the vms destroyed.

setVmsDestroyed(int) - Method in class org.cloudbus.cloudsim.network.datacenter.NetDatacenterBroker
:   Sets the vms destroyed.

setVmSelectionPolicy(PowerVmSelectionPolicy) - Method in class org.cloudbus.cloudsim.power.PowerVmAllocationPolicyMigrationAbstract
:   Sets the vm selection policy.

setVmsMigratingIn(List<String>) - Method in class org.cloudbus.cloudsim.VmScheduler
:   Sets the vms migrating in.

setVmsMigratingOut(List<String>) - Method in class org.cloudbus.cloudsim.VmScheduler
:   Sets the vms in migration.

setVmsRequested(int) - Method in class org.cloudbus.cloudsim.DatacenterBroker
:   Sets the vms requested.

setVmsRequested(int) - Method in class org.cloudbus.cloudsim.network.datacenter.NetDatacenterBroker
:   Sets the vms requested.

setVmsToDatacentersMap(Map<Integer, Integer>) - Method in class org.cloudbus.cloudsim.DatacenterBroker
:   Sets the vms to datacenters map.

setVmsToDatacentersMap(Map<Integer, Integer>) - Method in class org.cloudbus.cloudsim.network.datacenter.NetDatacenterBroker
:   Sets the vms to datacenters map.

setVmTable(Map<String, Host>) - Method in class org.cloudbus.cloudsim.network.datacenter.NetworkVmAllocationPolicy
:   Sets the vm table.

setVmTable(Map<String, Host>) - Method in class org.cloudbus.cloudsim.VmAllocationPolicySimple
:   Sets the vm table.

setWorkflowEngineId(int) - Method in class org.workflowsim.WorkflowScheduler
:   Sets the workflow engine id

shutdownEntity() - Method in class org.cloudbus.cloudsim.core.CloudInformationService
:   Shutdowns the CloudInformationService.

shutdownEntity() - Method in class org.cloudbus.cloudsim.core.CloudSimShutdown


shutdownEntity() - Method in class org.cloudbus.cloudsim.core.SimEntity
:   This method is invoked by the `Simulation` before the simulation finishes.

shutdownEntity() - Method in class org.cloudbus.cloudsim.Datacenter


shutdownEntity() - Method in class org.cloudbus.cloudsim.DatacenterBroker


shutdownEntity() - Method in class org.cloudbus.cloudsim.examples.CloudSimExample8.GlobalBroker


shutdownEntity() - Method in class org.cloudbus.cloudsim.network.datacenter.NetDatacenterBroker


shutdownEntity() - Method in class org.cloudbus.cloudsim.network.datacenter.Switch


shutdownEntity() - Method in class org.workflowsim.ClusteringEngine


shutdownEntity() - Method in class org.workflowsim.WorkflowEngine


shutdownEntity() - Method in class org.workflowsim.WorkflowPlanner


shutdownEntity() - Method in class org.workflowsim.WorkflowScheduler
:   Terminate this entity (WorkflowScheduler)

signalShutdown(Collection<Integer>) - Method in class org.cloudbus.cloudsim.core.CloudInformationService
:   Sends a signal to all entity IDs mentioned in the given list.

SIM\_ANY - Static variable in class org.cloudbus.cloudsim.core.CloudSim
:   A standard predicate that matches any event.

SIM\_NONE - Static variable in class org.cloudbus.cloudsim.core.CloudSim
:   A standard predicate that does not match any events.

SimEntity - Class in org.cloudbus.cloudsim.core
:   This class represents a simulation entity.

SimEntity(String) - Constructor for class org.cloudbus.cloudsim.core.SimEntity
:   Creates a new entity.

SimEvent - Class in org.cloudbus.cloudsim.core
:   This class represents a simulation event which is passed between the entities in the simulation.

SimEvent() - Constructor for class org.cloudbus.cloudsim.core.SimEvent
:   Create a blank event.

SIMULATION\_LIMIT - Static variable in class org.cloudbus.cloudsim.examples.power.Constants


size() - Method in class org.cloudbus.cloudsim.core.DeferredQueue
:   Returns the size of this event queue.

size() - Method in class org.cloudbus.cloudsim.core.FutureQueue
:   Returns the size of this event queue.

sort(List<T>) - Static method in class org.cloudbus.cloudsim.lists.CloudletList
:   Sorts the Cloudlets in a list based on their lengths.

sortByCpuUtilization(List<T>) - Static method in class org.cloudbus.cloudsim.power.lists.PowerVmList
:   Sort by cpu utilization.

SPACE\_SHARED - Static variable in class org.cloudbus.cloudsim.DatacenterCharacteristics
:   Spaced-shared system using First Come First Serve (FCFS) algorithm.

stages - Variable in class org.cloudbus.cloudsim.network.datacenter.NetworkCloudlet


start(String, String, VmAllocationPolicy) - Method in class org.cloudbus.cloudsim.examples.power.RunnerAbstract
:   Starts the simulation.

start(String) - Static method in class org.cloudbus.cloudsim.util.ExecutionTimeMeasurer
:   Start.

START\_SIMULATION - Static variable in class org.workflowsim.WorkflowSimTags


startEntity() - Method in class org.cloudbus.cloudsim.core.CloudInformationService
:   Starts the CloudInformationService entity.

startEntity() - Method in class org.cloudbus.cloudsim.core.CloudSimShutdown


startEntity() - Method in class org.cloudbus.cloudsim.core.SimEntity
:   This method is invoked by the `Simulation` class when the simulation is started.

startEntity() - Method in class org.cloudbus.cloudsim.Datacenter


startEntity() - Method in class org.cloudbus.cloudsim.DatacenterBroker


startEntity() - Method in class org.cloudbus.cloudsim.examples.CloudSimExample8.GlobalBroker


startEntity() - Method in class org.cloudbus.cloudsim.network.datacenter.NetDatacenterBroker


startEntity() - Method in class org.cloudbus.cloudsim.network.datacenter.Switch


startEntity() - Method in class org.workflowsim.ClusteringEngine


startEntity() - Method in class org.workflowsim.WorkflowEngine


startEntity() - Method in class org.workflowsim.WorkflowPlanner


startEntity() - Method in class org.workflowsim.WorkflowScheduler
:   Start this entity (WorkflowScheduler)

startSimulation() - Static method in class org.cloudbus.cloudsim.core.CloudSim
:   Starts the execution of CloudSim simulation.

starttime - Variable in class org.cloudbus.cloudsim.network.datacenter.NetworkCloudlet


StaticScheduler - Class in org.workflowsim.scheduler
:   Static algorithm.

StaticScheduler() - Constructor for class org.workflowsim.scheduler.StaticScheduler


stDev(List<Double>) - Static method in class org.cloudbus.cloudsim.util.MathUtil
:   Gets the standard deviation.

stopSimulation() - Static method in class org.cloudbus.cloudsim.core.CloudSim
:   Stops Cloud Simulation (based on `Simulation#runStop()`).

Storage - Interface in org.cloudbus.cloudsim
:   An interface which defines the desired functionality of a storage system in a Data Cloud.

submitCloudletList(List<? extends Cloudlet>) - Method in class org.cloudbus.cloudsim.DatacenterBroker
:   This method is used to send to the broker the list of cloudlets.

submitCloudletList(List<? extends NetworkCloudlet>) - Method in class org.cloudbus.cloudsim.network.datacenter.NetDatacenterBroker
:   This method is used to send to the broker the list of cloudlets.

submitCloudletList(List<? extends Cloudlet>) - Method in class org.workflowsim.WorkflowEngine
:   This method is used to send to the broker the list of cloudlets.

submitCloudlets() - Method in class org.cloudbus.cloudsim.DatacenterBroker
:   Submit cloudlets to the created VMs.

submitCloudlets() - Method in class org.workflowsim.WorkflowScheduler
:   Submit cloudlets (jobs) to the created VMs.

submitJobs() - Method in class org.workflowsim.WorkflowEngine
:   Submit jobs to the created VMs.

submitTaskList(List<Task>) - Method in class org.workflowsim.ClusteringEngine
:   This method is used to send to the broker the list of cloudlets.

submittime - Variable in class org.cloudbus.cloudsim.network.datacenter.NetworkCloudlet


submitVmList(List<? extends Vm>) - Method in class org.cloudbus.cloudsim.DatacenterBroker
:   This method is used to send to the broker the list with virtual machines that must be
    created.

submitVmList(List<? extends Vm>) - Method in class org.cloudbus.cloudsim.network.datacenter.NetDatacenterBroker
:   This method is used to send to the broker the list with virtual machines that must be
    created.

submitVmList(List<? extends Vm>, int) - Method in class org.workflowsim.WorkflowEngine
:   This method is used to send to the broker the list with virtual machines
    that must be created.

submitVmList(List<? extends Vm>) - Method in class org.workflowsim.WorkflowEngine


SUCCESS - Static variable in class org.cloudbus.cloudsim.Cloudlet
:   The Cloudlet has been executed successfully.

sum(List<? extends Number>) - Static method in class org.cloudbus.cloudsim.util.MathUtil
:   Sums a list of numbers.

sw - Variable in class org.cloudbus.cloudsim.network.datacenter.NetworkHost


Switch - Class in org.cloudbus.cloudsim.network.datacenter


Switch(String, int, NetworkDatacenter) - Constructor for class org.cloudbus.cloudsim.network.datacenter.Switch


switching\_delay - Variable in class org.cloudbus.cloudsim.network.datacenter.Switch


SwitchingDelayAgg - Static variable in class org.cloudbus.cloudsim.network.datacenter.NetworkConstants


SwitchingDelayEdge - Static variable in class org.cloudbus.cloudsim.network.datacenter.NetworkConstants


SwitchingDelayRoot - Static variable in class org.cloudbus.cloudsim.network.datacenter.NetworkConstants


Switchlist - Variable in class org.cloudbus.cloudsim.network.datacenter.NetworkDatacenter

A B C D E F G H I J L M N O P Q R S T U V W Z

- Overview
- Package
- Class
- Use
- Tree
- Deprecated
- Index
- Help

- Prev Letter
- Next Letter

- Frames
- No Frames

- All Classes
